# RELATIONSHIP OF CARDIOPULMONARY EXERCISE TESTING AND FIELD WALKING TESTS IN MILD-MODERATE ALZHEIMER’S DEMENTIA

**DOI:** 10.1093/geroni/igz038.2275

**Published:** 2019-11-08

**Authors:** Dereck L Salisbury, Fang Yu

**Affiliations:** University of Minnesota School of Nursing, Minneapolis, Minnesota, United States

## Abstract

The purpose of this study was to investigate relationships among peak exercise parameters on 6-minute walk (6MWT) and shuttle walk tests (SWT), and laboratory-based cardiopulmonary exercise testing (CPET). These relationships have been established in cardiopulmonary patient populations, but not in community-dwelling older adults with mild-moderate Alzheimer’s dementia (AD). This study is a cross-sectional analysis of the baseline data of 6MWT, SWT, and CPET from the FIT-AD Trial (n=88: 49 males [76.6 {7.0} years and MMSE 21.5{3.5}] and 39 females [77.3 {6.5} years and MMSE 22.1 {3.4}]). Peak values for each test included heart rate (HR), systolic blood pressure (SBP), and rating of perceived exertion (RPE). Peak oxygen assumption (VO2) was measured in the CPET. Peak walking distance (PWD) was measured for the 6MWT and SWT. CPET produced significantly higher peak HR (118.7 [17.5] vs. 106 [22.8] vs. 106 [18.8] bpm), RPE (16 [2.1] vs. 12 [2.3] vs. 11 [2.1]) and SBP (182 [23.7] vs. 156 [18.9] vs. 150 [16.9] mmHg) compared to the SWT and 6MWT respectively. PWD on SWT (240.4 [128.1] m) and 6MWT (364.3 [108.5] m) significantly correlated with peak VO2 (17.0 [4.3]ml/kg/min) on CPET (r=.44 and r=.43) respectively. Correlations of peak VO2 and PWD on SWT in persons with AD are considerably lower than what is seen for persons with cardiopulmonary diseases. This lower correlation seen in our sample may be due to shorter PWD on walking tests. Future research should focus how mobility affects correlation of peak values on these tests.

